# Further Understanding of Degradation Pathways of Microcystin-LR by an Indigenous *Sphingopyxis* sp. in Environmentally Relevant Pollution Concentrations

**DOI:** 10.3390/toxins10120536

**Published:** 2018-12-14

**Authors:** Qin Ding, Kaiyan Liu, Kai Xu, Rongli Sun, Juan Zhang, Lihong Yin, Yuepu Pu

**Affiliations:** Key Laboratory of Environmental Medicine Engineering, Ministry of Education of China, School of Public Health, Southeast University, Nanjing 210009, China; dingqin@seu.edu.cn (Q.D.); 220183438@seu.edu.cn (K.L.); 230189311@seu.edu.cn (K.X.); 101012172@seu.edu.cn (R.S.); 101011288@seu.edu.cn (J.Z.); lhyin@seu.edu.cn (L.Y.)

**Keywords:** MC-LR, environmental concentration, metabolite, degradation pathway, UPLC-MS/MS

## Abstract

Microcystin-LR (MC-LR) is the most widely distributed microcystin (MC) that is hazardous to environmental safety and public health, due to high toxicity. Microbial degradation is regarded as an effective and environment-friendly method to remove it, however, the performance of MC-degrading bacteria in environmentally relevant pollution concentrations of MC-LR and the degradation pathways remain unclear. In this study, one autochthonous bacterium, *Sphingopyxis* sp. m6 which exhibited high MC-LR degradation ability, was isolated from Lake Taihu, and the degrading characteristics in environmentally relevant pollution concentrations were demonstrated. In addition, degradation products were identified by utilizing the full scan mode of UPLC-MS/MS. The data illustrated that strain m6 could decompose MC-LR (1–50 μg/L) completely within 4 h. The degradation rates were significantly affected by temperatures, pH and MC-LR concentrations. Moreover, except for the typical degradation products of MC-LR (linearized MC-LR, tetrapeptide, and Adda), there were 8 different products identified, namely, three tripeptides (Adda-Glu-Mdha, Glu-Mdha-Ala, and Leu-MeAsp-Arg), three dipeptides (Glu-Mdha, Mdha-Ala, and MeAsp-Arg) and two amino acids (Leu, and Arg). To our knowledge, this is the first report of Mdha-Ala, MeAsp-Arg, and Leu as MC-LR metabolites. This study expanded microbial degradation pathways of MC-LR, which lays a foundation for exploring degradation mechanisms and eliminating the pollution of microcystins (MCs).

## 1. Introduction

The outbreak of harmful cyanobacterial blooms in eutrophic freshwater worldwide is a threat to aquatic ecosystem safety and public health, due to a series of toxic secondary metabolites that are produced [1,2,3]. Microcystins (MCs) are monocyclic heptapeptide cyanotoxins, with the typical structure of cyclo-(Ala-X-MeAsp-Z-Adda-Glu-Mdha) [4]. Due to demethylated or acetylated amino acid residues and variable L-amino acids at two non-conservative positions (X and Z), more than 100 analogs have been identified in the environment [5]. Most MCs can cause health risks to aquatic organisms and human beings through the inhibition of protein phosphatases 1 and 2A (PP1/2A) [6]. Furthermore, exposure of these MCs may lead to liver and kidney damage, reproductive toxicity, and even promotion of tumorigenesis [7,8,9,10]. Additionally, MCs can accumulate in the tissues of aquatic organisms, and exhibit a biological amplification effect through the aquatic food net, which seriously threatens human bodies [8,11,12,13]. Microcystin-LR (MC-LR, Figure S1), which has leucine (L) at position X and arginine (R) at position Z, is the most toxic and abundant among the MCs [14,15]. Therefore, to reduce the hazard caused by MC-LR, degradation in the field environment is necessary.

Studies have demonstrated that biological approaches may be the safest and most effective methods to remove dissolved MCs from natural water bodies by assimilating or eliminating the toxins without generating any harmful metabolites [16].Various biological methods have been proposed to eliminate hazardous MC-LR in freshwater, including the application of bioreactors, activated sludge and other biological treatments [17,18,19]. All of these typical methods are related to the bacterial degradation of MCs, so it is essential to explore the knowledge of bacterial degradation of MCs.

To date, dozens of microcystin (MC) degradable bacteria have been reported, including *Proteobacteria*, *Firmicutes*, and *Actinobacteria* [20,21]. However, most of these studies evaluated the maximum removal capacity and characteristics of functional bacteria at high levels of MC-LR (over 50 μg/L), but hardly at low concentrations of MC-LR (1–50 μg/L) [22]. In practice, low concentrations of MC-LR were closer to real environmental pollution levels compared to high MC-LR concentrations [23,24,25]. Previous studies indicated that the highest MC-LR concentration in the water of Lake Taihu was 16.23 μg/L, and 168.1 μg/kg in surface sediment during the outbreak of cyanobacterial blooms [23,24]. Major et al. even reported that the total MCs concentration reaches 33 μg/L in the Koka reservoir of Ethiopia [25]. But the specific performance of efficient MC-degrading bacteria in environmentally relevant pollution concentrations of MC-LR is unclear. The canonical MC-degradation pathway was acknowledged by *mlr* gene cluster that can express Mlr enzymes to sequentially hydrolyze the peptide bonds of MC-LR and intermediate products [14,26]. Apart from *mlr* gene cluster, some alternative degradation pathways were reported. Edwards et al. found demethylation, hydrolysis, decarboxylation and condensation of microcystin LF (MC-LF) and nodularin as new intermediate degradation products [27]. Ame et al. reported *Sphingomonas* sp. (CBA4) can demethylate microcystin RR (MC-RR), and the intermediate was degraded finally [28]. Dziga et al. detected four products of dmMC-LR, including cyclic dmMC-LR, two cyclic dmMC-LR with different modifications in the Arg-Asp-Leu region, and the tetrapeptide in temperate freshwater bodies [29]. Zhang et al. found that *Sphingopyxis* sp. USTB-05 can degrade MC-RR through hydrolysis and dehydration reaction to form a linear MC-RR with two small peptide rings [30]. However, the specific degradation products and their formation order were not explained thoroughly [31,32]. So it is important to analyze the specific behaviors and mechanism of MC-degrading bacteria in environmentally relevant pollution concentrations.

In this study, one bacterium with high MC-LR degrading ability was successfully isolated from the surface water of Lake Taihu, which is the third largest freshwater lake in China. The degrading performance of this bacterium was determined under various environmental factors, including different temperatures, pH, and MC-LR concentrations at environmentally relevant pollution levels. Furthermore, the metabolites and expression profiles of the *mlr* genes were analyzed during the degradation of MC-LR in environmentally relevant pollution concentrations. Moreover, through the discovery of new intermediate degradation products and the expression profiles of *mlr* genes during the degradation process, the possible pathways associated with MC-LR degradation were speculated.

## 2. Results

### 2.1. Bacterial Identification and the Maximum MC- LR Degrading Capability

**Figure 1:**
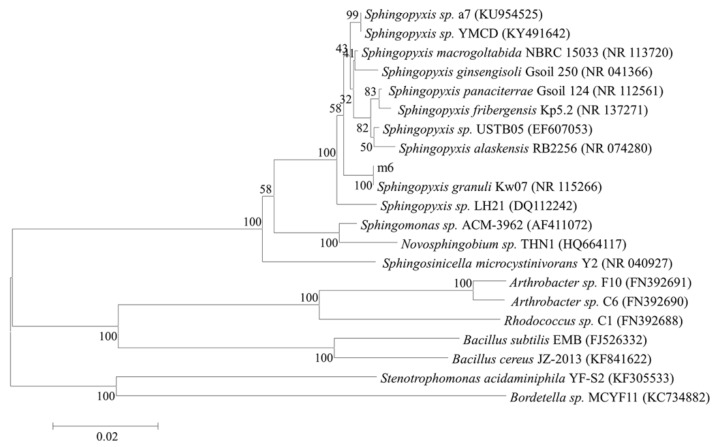
Phylogenetic tree of strain m6 based on bacterial 16S rDNA sequences with a neighbor-joining method (1000 bootstrap replicates). The scale bar represents 0.02 substitutions per nucleotide position and the branch numbers indicate bootstrap support. Accession numbers of reference sequences in Genbank are presented behind the strain names.

A bacterium with strong MC-LR degrading ability was isolated and denominated m6. The BLAST of the National Center for Biotechnology Information database (NCBI, www.ncbi.nlm.nih.gov) was used to retrieve the 20 most similar sequences, including the sequences of MC-degrading bacteria and the alignment was performed to construct a phylogenetic tree using the neighbor-joining analysis of software MEGA5.1. In the phylogenetic tree, this strain was tentatively identified as *Sphingopyxis* sp. m6, because of the high bootstrap value (Figure 1). The sequence analysis revealed that the maximum homologous similarity of 16S rDNA was 97% compared with *Sphingopyxis granuli* strain Kw07. The 16S rDNA nucleotide sequence had been deposited in the Genbank database under accession number MF535105.

**Figure 2:**
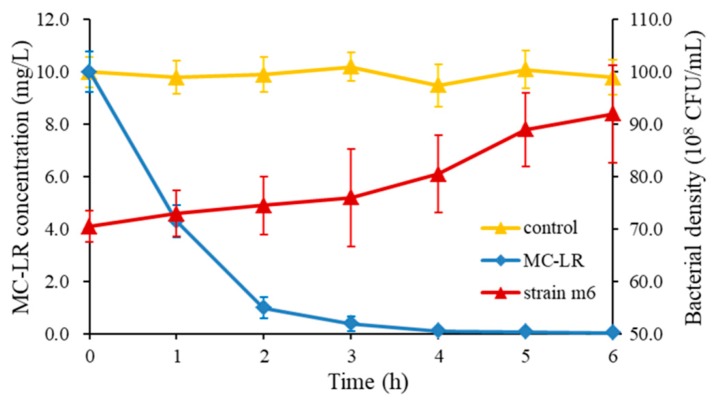
Degradation curve of MC-LR and growth curve of strain m6 at 10 mg/L MC-LR (30 °C, pH = 7). Bacteria-free culture served as the control. The values and error bars presented are the means and standard deviations (*n* = 3).

The MC-LR degradation curve and the growth curve of strain m6 (10 mg/L MC-LR, 30 °C, and pH = 7) are shown in Figure 2. Once strain m6 was added to the cultivation system, MC-LR was decomposed immediately without any lag phase. Almost all (99%) of the MC-LR was decomposed within 4 h. The average degradation rate was 60 mg/L/day, and the maximum degradation rate reached 136.3 mg/L/day in the first hour. Regarding the overall numbers of strain m6 during degradation, the bacterial number raised gradually from 7.05 × 10^9^ to 9.20 × 10^9^ CFU/mL. Through the entire degradation process, the growth rate of strain m6 remained stable during the first 3 h, increased rapidly from the third to the fifth hour, and then grew slowly in the fifth hour. It should be noted that no degradation of MC-LR was observed in the control (Figure 2). The standard curve of MC-LR quantitated by high performance liquid chromatography (HPLC) is shown in Figure S2.

### 2.2. MC-LR Degrading Activities in Environmentally Relevant Pollution Concentrations under Various Conditions

**Figure 3:**
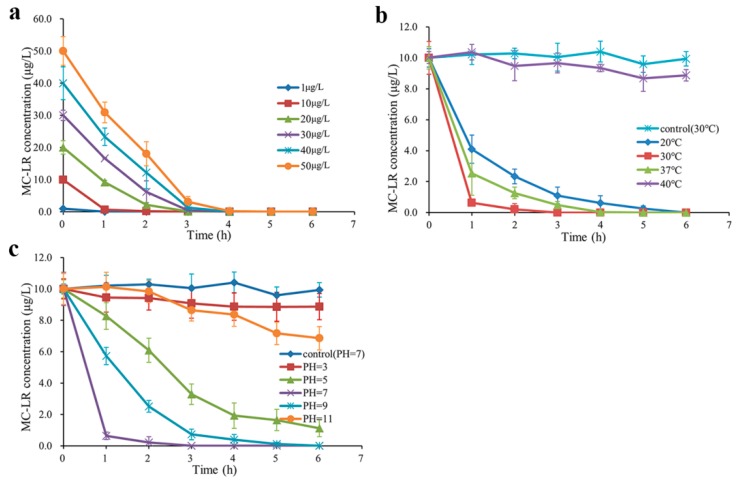
Effect of different conditions on the degradation rate of MC-LR by strain m6 in environmentally relevant pollution concentrations. (**a**) MC-LR concentration (30 °C, pH = 7), (**b**) Incubation temperature (pH = 7, 10 μg/L), and (**c**) pH (30 °C, 10 μg/L). The error bars indicate the standard deviation of three replicates.

Degradation experiments of MC-LR in environmentally relevant pollution concentrations were performed under different conditions (Figure 3, the standard curve of MC-LR quantitated by mass spectrometry is shown at Figure S3). Single factor experiments showed that degradation rates of MC-LR were affected by different MC-LR concentrations (Figure 3a), incubation temperatures (Figure 3b) and pH values (Figure 3c). Figure 3a illustrates that strain m6 degraded MC-LR at 1, 10, 20, 30, 40, and 50 μg/L with an average rate of 1.00, 3.33, 5.00, 7.50, 10.00, and 12.50 μg/L/h (30 °C, pH = 7), respectively. In Figure 3b, the results demonstrate that the average degradation rates of strain m6 were 1.67, 3.33, and 2.00 μg/L/h at 20, 30, and 37 °C (pH = 7, 10 μg/L), respectively. Furthermore, MC-LR was rarely degraded at 40 °C (Figure 3b). Figure 3c shows that at 30 °C, 10 μg/L MC-LR was decomposed by strain m6 at the average rate of 0.19, 1.48, 3.33, 1.67, and 0.52 μg/L/h at pH 3, 5, 7, 9, and 11, respectively. According to the results in Figure 3a, the shortest time required for thorough decomposition by *Sphingopyxis* sp. m6 was 1 h in 1 μg/L MC-LR, and the longest time was 4 h in 50 μg/L MC-LR (30 °C, pH = 7). The results indicate that the highest degrading rate of low MC-LR concentrations was 12.5 μg/L/h at 30 °C, pH = 7 and a concentration of 50 μg/L. There was no MC-LR catabolism in the control samples without strain m6.

### 2.3. Detection of Degradation Products of MC-LR

Twelve substances were detected in the different stages of degrading experiment (30 °C, pH = 7, and 50 μg/L MC-LR), including standard MC-LR and 11 intermediate degradation products (Table 1). Only MC-LR was eluted at 8.6 min at *m*/*z* 995.5545 ([M + H]^+^, C_49_H_75_N_10_O_12_) and 498.2815 ([M + 2H]^2+^, C_49_H_76_N_10_O_12_) in the samples of 0 h and standard MC-LR (Figures S4a, S5a). Linearized MC-LR (Ala-Leu-MeAsp-Arg-Adda-Glu-Mdha) was detected at 8.4 min with *m*/*z* of 507.2853 ([M + 2H]^2+^, C_49_H_78_N_10_O_13_) and 1013.5666 ([M + H]^+^, C_49_H_77_N_10_O_12_) (Figures S4b, S5b). One catabolite at *m*/*z* 615.3405 detected at 8.3 min was regarded as a tetrapeptide (Adda-Glu-Mdha-Ala, [M + H]^+^, C_32_H_47_N_4_O_8_) (Figures S4c, S5c). Three tripeptides (Adda-Glu-Mdha, Glu-Mdha-Ala, and Leu-MeAsp-Arg) were determined at *m*/*z* 544.3400 ([M + H]^+^, C_29_H_42_N_3_O_7_), 302.1354 ([M + H]^+^, C_12_H_20_N_3_O_6_), and 417.2458 ([M + H]^+^, C_17_H_33_N_6_O_6_) (Figures S4d-S4f, S5d-S5f). Three degradation products at *m*/*z* 231.1057, 173.0925, and 304.1619 were identical to Glu-Mdha ([M + H]^+^, C_9_H_15_N_2_O_5_), Mdha-Ala ([M + H]^+^, C_7_H_13_N_2_O_3_), and MeAsp-Arg ([M + H]^+^; C_11_H_22_N_5_O_5_), with retention times of 7.4, 4.9, and 1.3 min (Figures S4g-S4i, S5g-S5i). Single amino acids Adda, Leu, and Arg were also detected at *m*/*z* 332.2088 ([M + H]^+^, C_20_H_30_NO_3_), 132.1023 ([M + H]^+^, C_6_H_14_NO_2_), and 175.1202 ([M + H]^+^, C_6_H_15_N_4_O_2_) at 10.4, 3.4, and 1.3 min, respectively (Figures S4j-S4l, S5j-S5l). The *m*/*z* of above-detected degradation products does not match that of standard MC-LR fragment ions (Figure S6), which is 18 more than the corresponding fragment ions (one molecular weight of H_2_O). Based on the retention time of 11 intermediate substances in this study were different from that of MC-LR (Table 1), these metabolites are determined from the bacterial degradation of MC-LR and not the fragmentation of the toxin, due to mass spectrometry.

**Figure 4:**
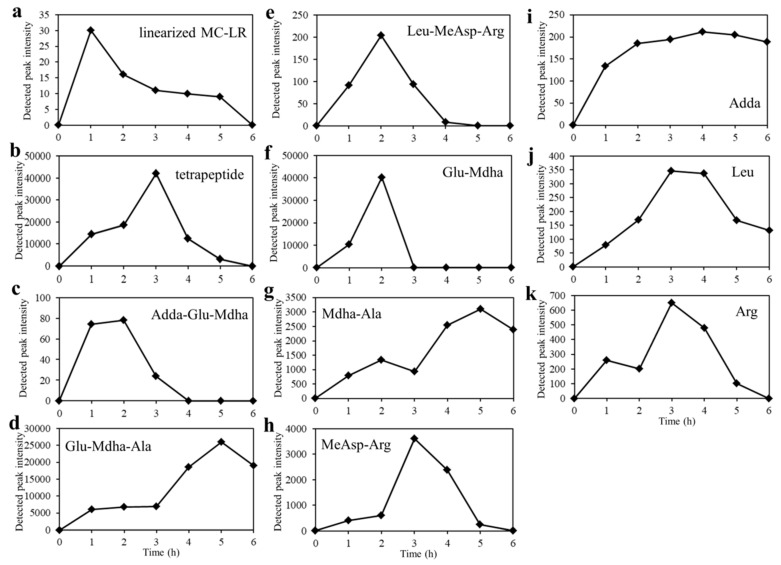
Degradation process of products by strain m6 based on the detected peak intensity. (**a**) linearized MC-LR, (**b**) tetrapeptide, (**c**) Adda-Glu-Mdha, (**d**) Glu-Mdha-Ala, (**e**) Leu-MeAsp-Arg, (**f**) Glu-Mdha, (**g**) Mdha-Ala, (**h**) MeAsp-Arg, (**i**) Adda, (**j**) Leu, (**k**) Arg.

Figure 4 illustrates the degradation process of all detected products from 0 h to 6 h using tandem mass spectrometry. The production of the linearized MC-LR was increasing rapidly to the maximum during the first hour and the tetrapeptide reached the maxima at 3 h. The amount of Leu-MeAsp-Arg, MeAsp-Arg, Leu, and Arg all presented a downward trend after reaching the maximum. Leu-MeAsp-Arg reached the maximum at 2 h and others needed 3 h. No product was detected in the samples of 50 μg/L standard MC-LR.

### 2.4. MC-Degrading Genes and Their Expression Profiles

Agarose gel electrophoresis of *mlr*-gene PCR products was performed, and four bright bands were observed after amplification. The fragment size was about 750, 1400, 1500, and 1100 bp, corresponding to the key enzyme genes *mlrA*, *mlrB*, *mlrC*, and *mlrD*, respectively. The sequences of *mlrA*, *B*, *C*, and *D* of strain m6 have the similarity of 99%, 98%, 100%, and 100% with the genes of *Sphingomonas* sp. USTB-05, *Sphingopyxis* sp. C-1, *Sphingomonas* sp. USTB-05, and *Sphingopyxis* sp. MB-E, respectively. The nucleotide sequences of *mlrA*, *B*, *C*, and *D* are available in the Genbank database with accession number MK179284–MK179287.

**Figure 5:**
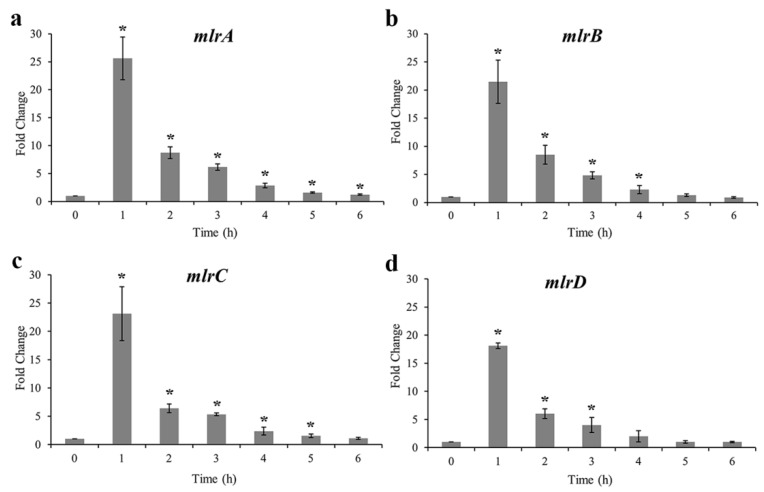
Expression profiles of genes *mlrA*, *mlrB*, *mlrC* and *mlrD* during the MC-LR degradation. (**a**) *mlrA* gene (**b**) *mlrB* gene (**c**) *mlrC* gene (**d**) *mlrD* gene. Asterisks represented those samples is statistically significant compared with that of the control (*n* = 3).

The expressions of four *mlr* genes during the degradation of MC-LR are shown in Figure 5. Similar expression profiles of the four genes can be observed, with a rapid increase in the first hour then a gradual decline to 1 fold from 2 h to 6 h. In the first hour, *mlrA* had the maximum 25-fold upregulation, and *mlrD* had the minimum 18-fold among the four *mlr* genes. The expression level of *mlrA* exhibited the slowest decrease, followed by *mlrC*, and *mlrD* decreased most rapidly.

## 3. Discussion

Biodegradation of MCs is an efficient and promising method to quickly remove them from natural water bodies and eliminate toxicity without the generation of toxic by-products, such as sand filters and pure bacterium. [4,33,34]. Members of the genus *Sphingopyxis* widely coexist in the natural aquatic ecosystem, and most of which have been connected with biodegrading complex organic matters, due to their tolerance of extreme poor nutrition through utilizing various simple molecules, especially aromatic compounds and biotoxins [35,36,37,38]. The first MC-degrading bacterium was identified in 1994 and the publications about bacterial degradation of MCs were updated all the times [20,39]. The degradation capability of these strains was varied from 1.5 μg/L/day to 29.5 mg/L/day [20,40,41]. However, their degradation rates were much lower than that of *Sphingopyxis* sp. m6 (60 mg/L/day). MC-LR was degraded rapidly without any lag phase once strain m6 was added (Figure 2). Low adaption of bacteria and abundance may be the reasons for the lag phase before the biodegradation of MC-LR [42,43]. The bacterial density of m6 maintained moderate growth in the first 3 h and then rapidly increased after MC-LR was decomposed (Figure 2). The number of degradation products increased with the decomposition of MC-LR, and the former may be easily assimilated as a carbon and nitrogen source for strain m6 growth. Varied strain characteristics were probably attributed to their different species or unique functional genes.

Many bacteria with high MC-degrading ability were separated in individual studies all over the world [21]. However, there were few application tests in MC-LR of environmentally relevant pollution concentrations. MC-LR degradation rates of strain m6 were significantly affected by temperatures, pH, and MC-LR concentrations in environmentally relevant pollution concentrations (Figure 3a–c). The optimized degradation conditions occurred at the toxin concentration of 50 μg/L, 30 °C, and pH = 7. The degradation rates accelerated as the increase of MC-LR in environmentally relevant pollution concentrations, which was in agreement with the phenomenon in high concentration MC-LR (Figure 3a) [44]. Temperature played a crucial role in the microbial degradation, with the highest degradation rate at optimum temperature and a rapid decrease, due to an increase or decline in temperature (Figure 3b) [45,46]. MC-degrading enzymes may be sensitive to environmental temperature, and this probably explained the higher MC levels of the freshwater ecosystem in the summer [47]. Most of the water bodies differ in pH values during cyanobacterial blooms [48,49]. Neutral or weak alkaline conditions were more suitable to work for strain m6, which is consistent with previous studies (Figure 3c) [34,49].

**Figure 6:**
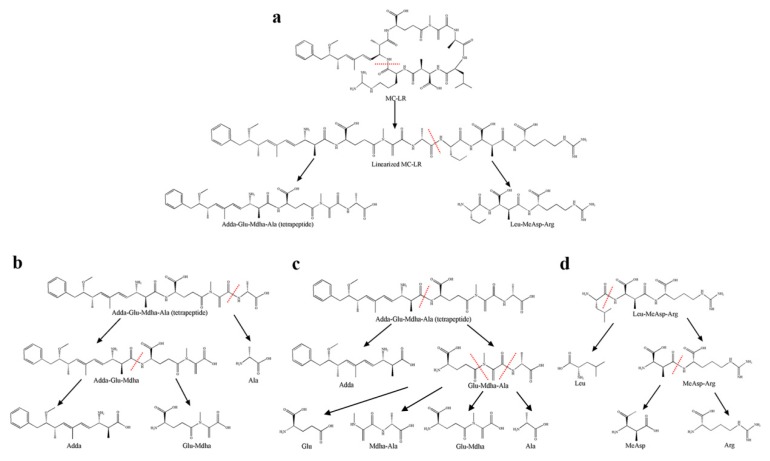
Proposed biodegradation pathways of MC-LR by strain m6. (**a**) The degradation procedure from MC-LR to tetrapeptide, (**b**,**c**) two different degradation processes of tetrapeptide, (**d**) the degradation process of Leu-MeAsp-Arg. The dotted line showed the cleavage positions of the peptide bonds.

In this study, 11 intermediate degradation products had been detected (Table 1), and according to the degradation process of products (Figure 4), the specific degradation pathways of MC-LR by *Sphingopyxis* sp. m6 are described in Figure 6. First, a linearized MC-LR was generated through the breaking of a peptide bond at the Adda-Arg to open the cyclo-heptapeptide [26,50]. The peptide bond at Ala-Leu was hydrolyzed, producing one tetrapeptide (Adda-Glu-Mdha-Ala) and one tripeptide (Leu-MeAsp-Arg) (Figure 6a) [26,51]. The above two-step enzymatic cleavage had already been reported in the previous literature [20,52]. The tetrapeptide was decomposed in two ways, as shown in Figure 6b,c. The cleavage of the peptide bond at Mdha-Ala and Adda-Glu of the tetrapeptide formed a tripeptide (Adda-Glu-Maha), Ala, and Adda, a tripeptide (Glu-Mdha-Ala), respectively. Successive cleavage of Adda-Glu in the tripeptide (Adda-Glu-Maha) generated Adda and a dipeptide (Glu-Mdha). Moreover, another tripeptide (Glu-Mdha-Ala) was decomposed into amino acid Glu and a dipeptide (Mdha-Ala), due to the cracking in bond Glu-Mdha, or degraded into dipeptide (Glu-Mdha) and Ala through the hydrolysis of the peptide bond in Mdha-Ala. On the other side, the counterpart tripeptide (Leu-MeAsp-Arg) was detached to a single amino acid Leu and a dipeptide (MeAsp-Arg), and then the dipeptide (MeAsp-Arg) was hydrolyzed to two amino acids, MeAsp and Arg synchronously (Figure 6d). It is well known that Adda is the main bioactive structure to determine the PP1/2A inhibition ability of MCs [33]. In this study, the Adda can be decomposed slowly, which indicated that *Sphingopyxis* sp. m6 can detoxicate the toxicity of MC-LR further than *Sphingomonas* sp. ACM-3962 and *Sphigopyxis* sp. C-1 in which Adda was the final degradation product of MCs [49,53]. The production of Adda was gradually increasing during the degradation of MC-LR by strain B-9 [32]. It was also shown that the presence of the other gene, which is responsible for the degradation of Adda in strain m6.

Bourne et al. first proposed that microcystinase MlrA hydrolyzed cyclic MC-LR to linearized MC-LR with breaking at Arg-Adda and a tetrapeptide was generated from linearized MC-LR by hydrolyzation of a peptide bond at Leu-Ala by MlrB [26]. MlrC can decompose both linearized MC-LR and tetrapeptide into Adda through the fracture of Adda-Glu [31,51]. MlrD was predicted as an oligopeptide transporter, due to the potential transmembrane spanning regions [53]. Therefore, the *mlr* genes of strain m6 and their expression profiles during the biodegradation were further analyzed in this study. All four of the *mlr* genes were determi ned and significantly upregulated in the first hour of degradation. Thereafter, there was a slow decline in expression fold changes (Figure 5). It was found that faster degradation rates occurred in higher initial concentrations of MC-LR (Figure 2 and Figure 3a). It was proved that Adda can induce the expression of *mlrA* and *mlrB* genes [31]. There was probably more existence of Adda residue with higher concentrations of MC-LR, and then Adda stimulated the higher expression of *mlrA* and *mlrB* genes to accelerate the decomposition of MC-LR or intermediates, correspondingly. This is likely to explain the relation between MC-LR concentrations and degradation rates. The ring opening was the first and most critical step in the degradation of MCs by enzyme MlrA, so the expression level and duration time of the *mlrA* gene was higher than that of the other three [50].

Due to the unavailability standards for these degradation products, as well as the response signal of mass spectrometer varying with different types of substances, it was difficult to quantitate the exact concentrations of metabolites [54]. The potential degradation pathways cannot be excluded due to the absence of other degradation products from LC-MS/MS. Therefore, only the detected products were used to infer the possible degradation pathways in this manuscript. In this study, the metabolites in Figure 6 were all detected, except for amino acids Glu, Ala, and MeAsp. Amino acid Ala with *m*/*z* 90.0550 was out of detection in this method, while Glu and MeAsp were probably degraded or assimilated quickly in the culture system. Dziga et al. reported degradation product hexapeptides, which were produced through MlrC hydrolyze the peptide bond at Adda-Glu of linearized MCs, and the hexapeptides were degraded into Glu-Mdha-Ala and X-MeAsp-Z by MlrB toward Ala-X bond presumably [31]. In this study, the hexapeptides were not detected, but Glu-Mdha-Ala and Leu-MeAsp-Arg existed. These intermediates were probably degraded by MlrB immediately into two tripeptides. Notably, two dipeptides (Mdha-Ala, and MeAsp-Arg) and Leu were identified initially in the metabolism pathways of MC-LR based on mass spectrometry analysis in this study [32]. Due to the existence of new metabolites, other new types of hydrolases may exist, or the original Mlr enzymes have multiple degradation functions to break peptide bonds at Leu-MeAsp, and Glu-Mdha. These new intermediates expand the microbial degradation pathways of MCs and lay the foundation for the biodegradation of MCs.

## 4. Conclusions

In this study, an autochthonous *Sphingopyxis* sp. m6 was obtained with strong degradation capability of MC-LR from Lake Taihu. MC-LR of environmentally relevant pollution concentrations (1–50 μg/L) was rapidly degraded by strain m6 under the optimal conditions. Strain m6 had *mlrA*, *B*, *C*, and *D* genes that were highly transcribed during the catabolism of MC-LR. The present study detailed the specifically hydrolytic pathways through the determination of intermediate products, which had never been reported and provided a basis for further studies on the microbial degradation mechanisms of MCs. It is highly significant to determine the catabolic capacity of other environmental contaminants containing peptide bonds by strain m6. Whether the original MC-degrading *mlr* gene clusters have multiple functions or some other degradation genes existed to produce and disintegrate new intermediates, should be further studied to accelerate solving these pollution problems in the aquatic ecosystem.

## 5. Materials and Methods

### 5.1. Standard Toxin and Reagents

Standard MC-LR with Purity ≥ 95% was obtained from Enzo Life Sciences Incorporation (Farmingdale, NY, USA). The mineral salt medium (MSM) used for the bacterial culture containing (g/L): K_2_HPO_4_ 4.0, MgSO_4_·7H_2_O 1.0, NaCl 1.0, KH_2_PO_4_ 0.5, CaCl_2_ 0.02, FeSO_4_ 0.005, MnCl_2_·4H_2_O 0.005, ZnCl_2_ 0.005, and CuCl_2_ 0.0005. The pH of MSM was adjusted to 7.0 and sterilized before use [55]. All of the chemical substances contained in MSM were purchased from Wanqing Chemical Co., Ltd. (Nanjing, China). Trifluoroacetic acid and methanol were purchased from Macklin Biochemical Co.,Ltd. (Shanghai, China) and Tedia Company, Inc. (Fairfield, OH, USA), respectively. Acetonitrile (Merck, Darmstadt, Germany) and formic acid (Fisher Scientific, Shanghai, China) were used for mass spectrometry.

### 5.2. Acquisition of a Functional Bacterium and Evaluation of MC-LR Degrading Capability

The water samples were collected from cyanobacteria salvage yards in Fudu bay, Taihu Lake, China, in July 2016. Ten milliliters of water was diluted 10 times by sterile lake water, then 1 mL supernatants were inoculated into 9 mL MSM in which 10 mg/L standard MC-LR was added as the sole carbon and nitrogen source (pH = 7, 30 °C, shaking at 120 rpm). The concentrations of MC-LR in the medium were detected by HPLC at intervals [21]. When the MC-LR was decomposed completely, 100 μL enriched cultures were serially diluted and spread on solid MSM (2% agar) supplemented with 10 mg/L MC-LR. Single colonies were picked up based on different morphology and inoculated into liquid MSM containing standard 10 mg/L MC-LR (pH = 7, 30 °C, shaking at 120 rpm) [55]. The medium without bacteria was cultured at the same condition as the control. One hundred microliter cultures were sampled from the culture system every 1 h, and 80 μL supernatants were transferred to autosamper vials after centrifugation (12,000× *g*, 15 min and 4 °C) for quantitative analysis of MC-LR. The number of the strain was counted by gradient dilution and culturing on lysogeny broth agar culture plates. The bacterium with the highest degradation ability was selected and designated as m6. All of the experiments were repeated three times.

The genomic DNA was extracted by a bacterial genomic DNA extraction kit (TaKaRa, Kusatsu, Japan) and polymerase cycle reaction (PCR) amplification of a bacterial 16S rDNA gene fragment was conducted using universal primers 27F, 1492R [56]. The PCR products were sequenced via the BGI Co., Ltd. (Shanghai, China), then DNA sequences were blasted in the NCBI database. Multiple sequence alignments and phylogenetic tree were constructed by comparing the sequences with similar 16S rDNA sequences from the NCBI database using the program clustalW and neighbor-joining analysis of software MEGA5.10 (2012) with 1000 bootstrap replications [21,57].

### 5.3. MC-LR Degradation Experiments under Environmentally Relevant Pollution Concentrations

The cells of strain m6 were harvested by centrifugation (5000× *g*, 15 min, 4 °C) and washed twice with 0.02 M phosphatic buffer solution after being cultured for 24 h with shaking at a constant condition (30 °C, 120 rpm) [21]. The collected cells were re-suspended and cultured into MSM containing standard MC-LR. The degradation experiments in simulated environmentally relevant pollution concentrations were performed under batch incubation conditions, including different MC-LR concentrations (1 μg/L, 10 μg/L, 20 μg/L, 30 μg/L, 40 μg/L, and 50 μg/L), temperature (20 °C, 30 °C, 37 °C, and 40 °C) and pH values (pH = 3, 5, 7, 9, and 11). One hundred microliter samples were withdrawn every 1 h and centrifuged (12,000× *g*, 15 min and 4 °C), then 80 μL supernatants were detected by tandem mass spectrometry for remaining MC-LR concentrations even degradation products in the co-incubation system immediately. Bacteria-free samples (10 μg/L MC-LR, 30 °C, and pH = 7) served as the control and all of the experiments were carried out three times.

### 5.4. Determination of MC-LR and Degradation Products

HPLC (Agilent 1100, Santa Clara, CA, USA) was used to analyze the concentrations of MC-LR over 50 μg/L with a Zorbax Extend C18 column (2.1 × 50 mm, particle size 1.8 µm, Agilent, Santa Clara, CA, USA) and a variable wavelength detector (VWD) at 238 nm. The mobile phase was the constant mixture of 0.05% trifluoroacetic acid aqueous solution and methanol (47:53, *v*/*v*), with a flow rate at 1 mL/min, injection volume of 20 μL, and column temperature of 40 °C.

Quantitative analysis of low concentrations of MC-LR (0.01–50 μg/L) was performed on the system of ultra performance liquid chromatography coupled with a tandem mass spectrometer (UPLC-MS/MS, triple TOF 5600+, AB Sciex, Redwood, CA, USA). The sample was separated by the UPLC system with an Acquity UPLC BEH C18 column (2.1 × 50 mm; particle size 1.7 μm; Waters, Milford, MA, USA) and the temperature of the column oven was maintained at 40 °C, and injection volume was 5 L. [55]. The mobile phases were acetonitrile and 0.1% formic acid aqueous solution: The organic phase was increased from 45% to 60% linearly in the first 3 min, then added to 100% in 0.1 min and held for 1 min, then returned to 45% and kept for 0.8 min until the next detection, and the flow rate was 0.2 mL/min. Mass spectrometry was operated in multiple-reaction monitoring (MRM) mode with positive mode and electrospray ionization. Parameters were Ionspray voltage floating of 5500 V, curtain gas of 35 psi, ion source gas 1, 2 of 50 psi, and interface heater temperature of 500 °C.

The identification of standard MC-LR (50 μg/L) and its degradation products by strain m6 in different degradation stages (50 μg/L, 30 °C, pH = 7) also used UPLC-MS/MS, which was equipped with an Acquity UPLC HSS T3 column (2.1 × 100 mm; particle size 1.8 μm; Waters, Milford, MA, USA). The gradient elution was 2% acetonitrile kept 2 min, then increased linearly to 98% within 12 min, held for 3 min and finally decreased to 2% and kept for 3 min. The signal data was acquired in information-dependent acquisition (IDA) acquisition mode from 100–1200 *m*/*z*. The flow rate was 0.3 mL/min and parameters settings of the mass spectrometer were ionspray voltage floating of 5000 V, declustering potential of 80 V, collision energy of 35 V, and collision energy spread of 15 V.

### 5.5. Detection of MC-Degrading Genes and Analysis of Their Expression Profiles

Genes *mlrA*, *mlrB*, *mlrC*, and *mlrD* were considered to be the main degrading genes of MCs in bacteria and were amplified with specific primers (Table S1) [58,59,60]. The PCR products were subsequently purified and sequenced by BGI Co., Ltd. (Shanghai, China). Sequences of *mlr* genes were deposited in the Genbank and then blasted with the similar sequences of the NCBI database.

Bacterial RNA of strain m6 in different degradation stages (50 μg/L, 30 °C, pH = 7) was extracted by TRIzol (Invitrogen, New York, NY, USA) and reverse transcribed immediately using PrimeScriptTM RT Master Mix (TaKaRa, Kusatsu, Japan), according to the instructions of manufacturer. Real-time PCR was executed by SYBR Green Real-time PCR Master Mix (Toyobo, Osaka, Japan) and the relative expression was analyzed by the 2^−ΔΔCt^ method. Primers 16S-F-real and 16S-R-real were used as the reference genes, and other primer sequences used are listed in Table S1 (designed by software Primer Premier 5.00, PREMIER Biosoft, Palo Alto, CA, USA). All of the samples were analyzed in triplicate.

### 5.6. Statistical Analysis

Data were shown as means ± standard deviations in figures, and the student’s *t*-test was applied to establish difference among samples. *p* < 0.05 was considered to be significant.

## Figures and Tables

**Table 1:** The detected degradation products of MC-LR.

Compound	Retention Time/min	Mass-to-Charge Ratio (*m*/*z*)		Predicted Structure
		Predicted	Detected	
MC-LR	8.6	995.5561	995.5545	cyclo(Ala-Leu-MeAsp-Arg-Adda-Glu-Mdha-H)
		498.2817	498.2815	cyclo(Ala-Leu-MeAsp-Arg-Adda-Glu-Mdha-2H)
linear MC-LR	8.4	1013.5666	1013.5666	Ala-Leu-MeAsp-Arg-Adda-Glu-Mdha-H
		507.2869	507.2853	Ala-Leu-MeAsp-Arg-Adda-Glu-Mdha-2H
tetrapeptide	8.3	615.3388	615.3405	Adda-Glu-Mdha-Ala-H
Adda-Glu-Mdha	11.8	544.3017	544.3400	Adda-Glu-Mdha-H
Glu-Mdha-Ala	4.9	302.1347	302.1354	Glu-Mdha-Ala-H
Leu-MeAsp-Arg	5.1	417.2456	417.2458	Leu-MeAsp-Arg-H
Glu-Mdha	7.4	231.0976	231.1057	Glu-Mdha-H
Mdha-Ala	4.9	173.0921	173.0925	Mdha-Ala-H
MeAsp-Arg	1.3	304.1616	304.1619	MeAsp-Arg-H
Adda	10.4	332.2220	332.2088	Adda-H
Leu	3.4	132.1019	132.1023	Leu-H
Arg	1.3	175.1190	175.1202	Arg-H

## Supplementary Materials

The following are available online at http://www.mdpi.com/2072-6651/10/12/536/s1, Table S1: Specific primers sequences; Figure S1: Chemical structure of MC-LR; Figure S2: The standard curve of MC-LR quantitated by HPLC (50 μg/L – 20 mg/L); Figure S3: The standard curve of MC-LR quantitated by UPLC-MS/MS (0.01 μg/L – 50μg/L); Figure S4: Chromatograms of MC-LR and its degradation products [(a) MC-LR, (b) linearized MC-LR, (c) tetrapeptide, (d) Adda-Glu-Mdha, (e) Glu-Mdha-Ala, (f) Leu-MeAsp-Arg, (g) Glu-Mdha, (h) Mdha-Ala, (i) MeAsp-Arg, (j) Adda, (k) Leu, (l) Arg]; Figure S5: Mass spectrum of MC-LR and its degradation products [(a) MC-LR, (b) linearized MC-LR, (c) tetrapeptide, (d) Adda-Glu-Mdha, (e) Glu-Mdha-Ala, (f) Leu-MeAsp-Arg, (g) Glu-Mdha, (h) Mdha-Ala, (i) MeAsp-Arg, (j) Adda, (k) Leu, (l) Arg]. Figure S6: Fragment ions of standard MC-LR detected by UPLC-MS/MS.
