# Supplementary material for: Further Understanding of Degradation Pathways of Microcystin-LR by an Indigenous Sphingopyxis sp. in Environmentally Relevant Pollution Concentrations
Source: Toxins (Basel). 2018 Dec 14;10(12):536. doi: 10.3390/toxins10120536 (PMC6315713; doi:10.3390/toxins10120536)
Supplement: Supplementary file 1 [file toxins-10-00536-s001.pdf]

Supplementary Materials: Further Understanding of Degradation Pathways of Microcystin-LR by an Indigenous *Sphingopyxis* sp. in Environmentally Relevant Pollution Concentrations

Qin Ding, Kaiyan Liu, Kai Xu, Rongli Sun, Juan Zhang, Lihong Yin, and Yuepu Pu

Table S1. Specific primers sequences.

Primers	Sequence (5' to 3')
mlrA-F	GACCCGATGTTCAAGATACT
mlrA-R	CTCCTCCCACAAATCAGGAC
mlrB-F	CTCGATGCGGTATTTGCTG
mlrB-R	TCCAACGACCATCCCTTCTG
mlrC-F	CGAAGGCGAAAGGTGCAAC
mlrC-R	GAGCGCTTGTGATAGTGACG
mlrD-F	GTTCCTCGGCGTAGCCT
mlrD-R	GCGACGAAGATCGTTGCT
16S-F-real	AGGATGAGCCCGCGTAAGAT
16S-R-real	TCAGTCCCAGTGTGGCTGATC
mlrA-F-real	GCACCATCATCGCTGTCTT
mlrA-R-real	ACCGTTGGAGCCCATTCG
mlrB-F-real	CTATCAGGGCGGCTTTGGA
mlrB-R-real	GAGAGCGGCCGTGAACTG
mlrC-F-real	CAGCGTCGATCGCACAAG
mlrC-R-real	CGCTCTGGGCCGTTACC
mlrD-F-real	ACGCCATCTTCTGCCTCC
mlrD-R-real	CGACCCGCAAGACGATTA

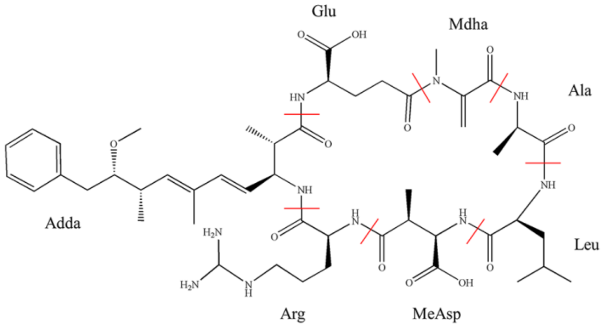

Figure S1. Chemical structure of MC-LR, cyclo-(Ala-Leu-MeAsp-Arg-Adda-Glu-Mdha).

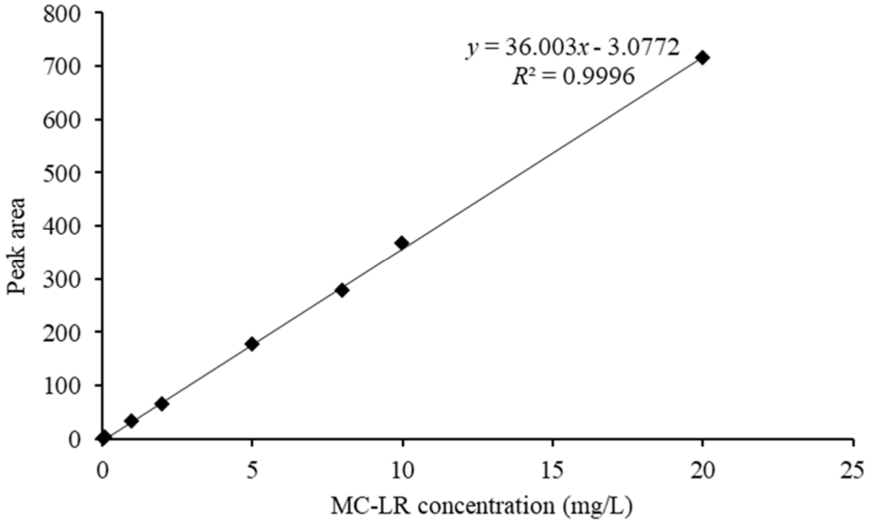

Figure S2. The standard curve of MC-LR quantitated by HPLC (50 μg/L–20 mg/L).

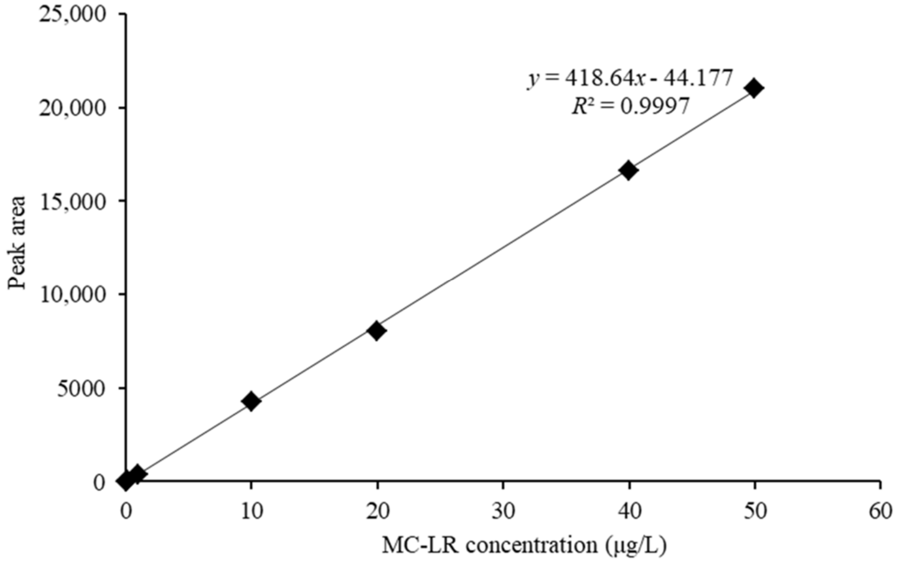

Figure S3. The standard curve of MC-LR quantitated by UPLC-MS/MS (0.01 μg/L–50μg/L).

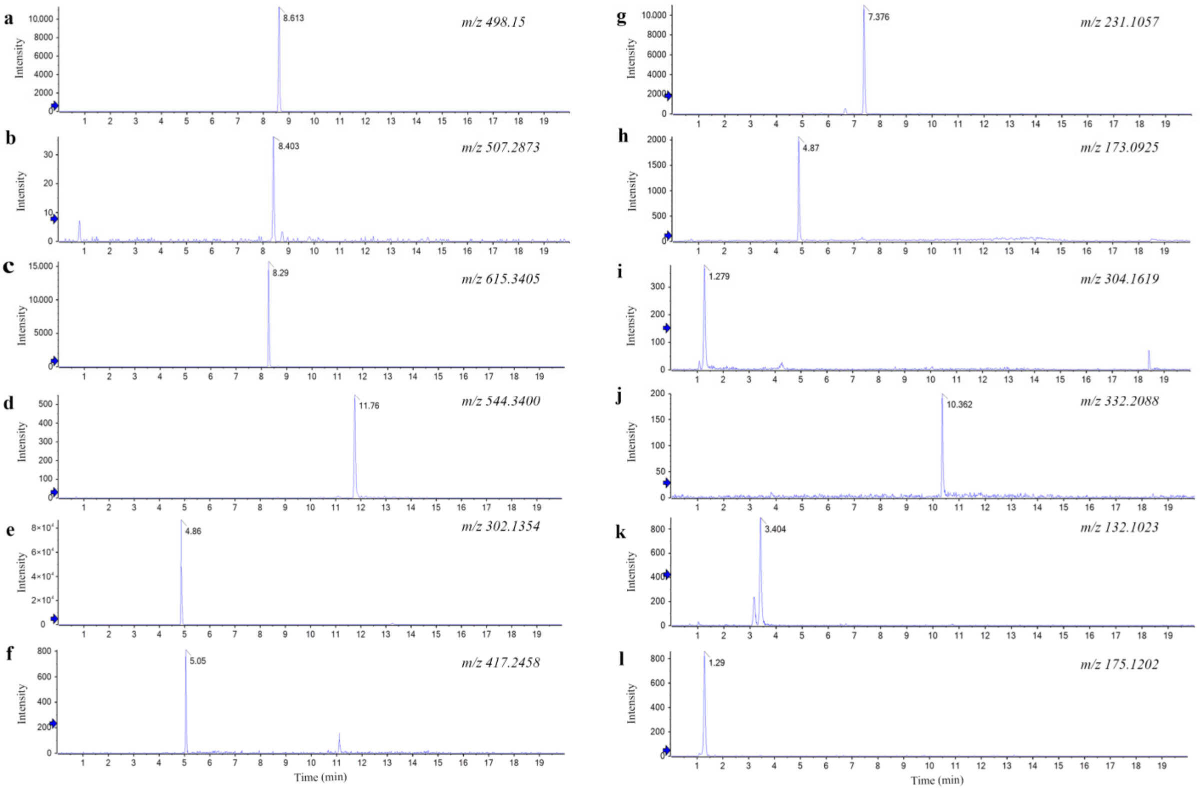

Figure S4. Chromatograms of MC-LR and its degradation products (**a**) MC-LR, (**b**) linearized MC-LR, (**c**) tetrapeptide, (**d**) Adda-Glu-Mdha, (**e**) Glu-Mdha-Ala, (**f**) Leu-MeAsp-Arg, (**g**) Glu-Mdha, (**h**) Mdha-Ala, (**i**) MeAsp-Arg, (**j**) Adda, (**k**) Leu, (**l**) Arg.

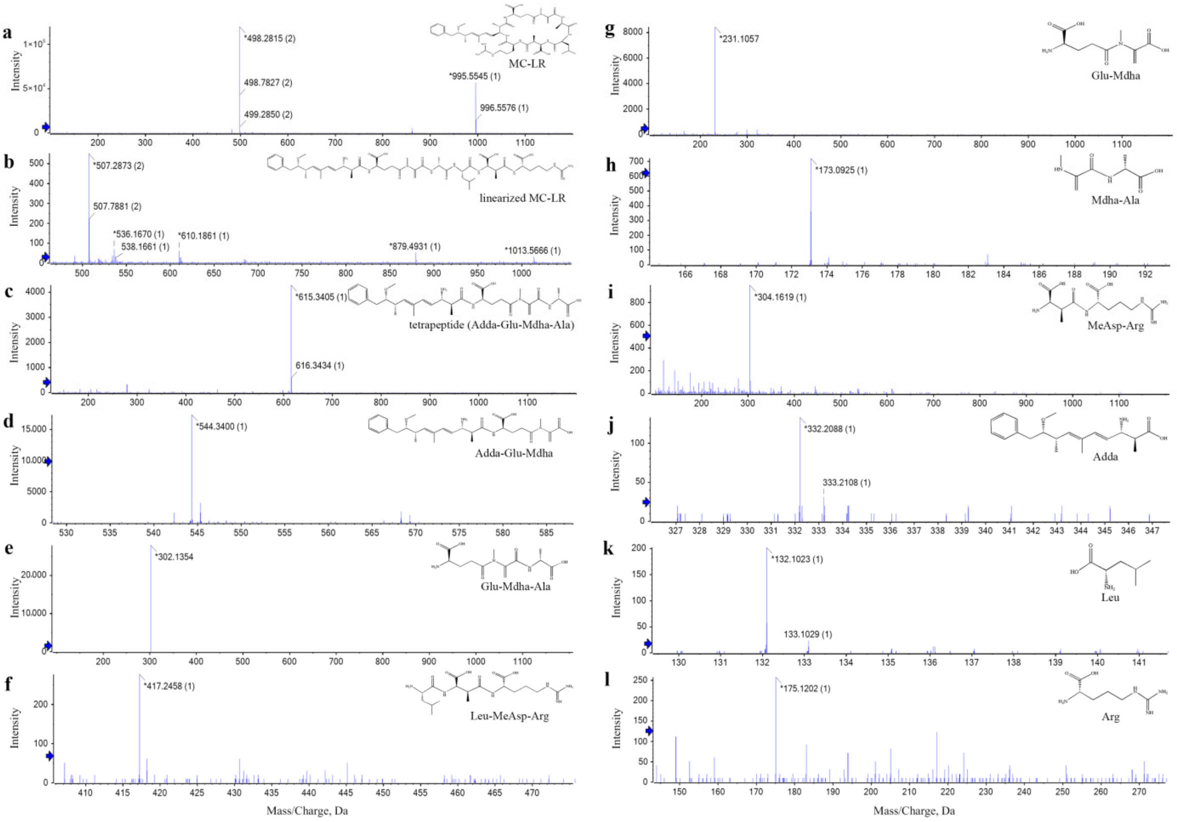

Figure S5. Mass spectrum of MC-LR and its degradation products (**a**) MC-LR, (**b**) linearized MC-LR, (**c**) tetrapeptide, (**d**) Adda-Glu-Mdha, (**e**) Glu-Mdha-Ala, (**f**) Leu-MeAsp-Arg, (**g**) Glu-Mdha, (**h**) Mdha-Ala, (**i**) MeAsp-Arg, (**j**) Adda, (**k**) Leu, (**l**) Arg.

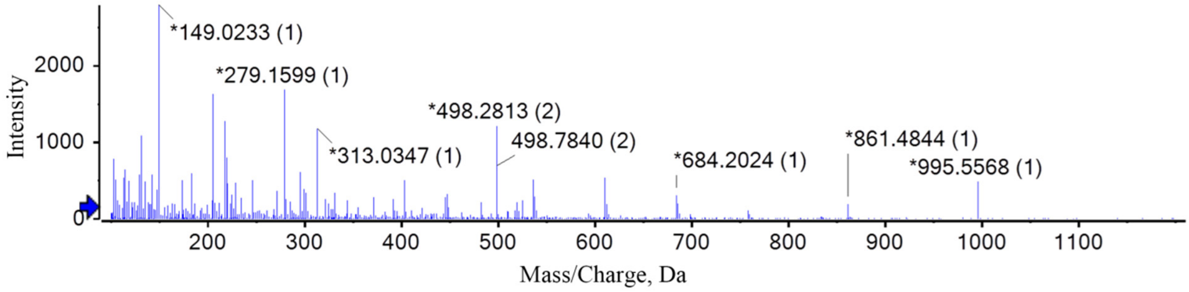

Figure S6. Fragment ions of standard MC-LR detected by UPLC-MS/MS.
